# A high performance N-doped graphene nanoribbon based spintronic device applicable with a wide range of adatoms†

**DOI:** 10.1039/d0na00652a

**Published:** 2020-11-09

**Authors:** M. Reza Rezapour, Geunsik Lee, Kwang S. Kim

**Affiliations:** Center for Superfunctional Materials, Department of Chemistry, Ulsan National Institute of Science and Technology (UNIST) Ulsan 44919 Korea kimks@unist.ac.kr gslee@unist.ac.kr

## Abstract

Designing and fabricating nanosize spintronic devices is a crucial task to develop information technology of the future. However, most of the introduced spin filters suffer from several limitations including difficulty in manipulating the spin current, incapability in utilizing a wide range of dopants to provide magnetism, or obstacles in their experimental realization. Here, by employing first principles calculations, we introduce a structurally simple and functionally efficient spin filter device composed of a zigzag graphene nanoribbon (ZGNR) with an embedded nitrogenated divacancy. We show that the proposed system, possessing a robust ferromagnetic (FM) ordering, exhibits perfect half metallic behavior in the absence of frequently used transition metals (TMs). Our calculations also show that the suggested system is compatible with a wide range of adatoms including basic metals, metalloids, and TMs. It means that besides d electron magnetism originating from TMs, p electrons of incorporated elements of the main group can also cause half metallicity in the electronic structure of the introduced system. Our system exploiting the robustness of doping-induced FM ordering would be beneficial for promising multifunctional spin filter devices.

## Abstract 2

Designing and fabricating nanosize spintronic devices is a crucial task to develop information technology of the future.

## Graphical abstract

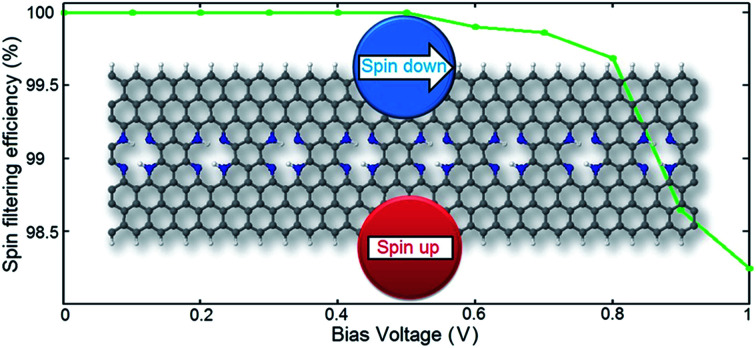

## 1. Introduction

Spintronics as a promising route for delivering, storing, and processing of information has attracted vast research attention due to its remarkable advantages for next-generation information technology.^1,2^ To fabricate commercial nanosize spintronic devices, developing experimentally feasible low-dimensional magnetic materials with high spin polarization is a necessity. In recent years, enormous effort has been devoted to exploring low-dimensional half metals, including various organic and inorganic two-dimensional (2D) sheets,^3–7^ as well as one dimensional (1D) nanowires.^8–12^ Experimental observation of 2D ferromagnets^13–15^ provided the motivation for recent theoretical studies which aim to investigate the possibility of realization of ferromagnetism in other 2D and 1D nanostructures.^16–19^ In these studied systems, magnetic properties are originated from the d electrons of the incorporated TM, and hence, give rise to disadvantages such as high-temperature processing and unavoidable spin relaxation caused by spin–orbit coupling. One possible solution for this issue is generating magnetism in p electrons of the main group elements. On the other hand, among low dimensional materials, graphene^20,21^ has attracted significant attention, both experimentally and theoretically, due to its extraordinary properties.^22–30^ Graphene in its 1D form, the so called graphene nanoribbon (GNR), has also been intensively investigated as a promising material for realization of nanoelectronics and spintronic nanodevices.^31–41^ These features are due to graphene's (hence GNRs') long spin relaxation and decoherence times originating from low intrinsic spin–orbit interactions and the low hyperfine interaction of the electron spins with the carbon nuclei in this material.^42–44^ Studies have shown the advent of magnetism in graphene based systems such as C3N4 nanosheets,^45^ nanographene,^46^ and graphene nanoribbons.^47^ The origin of the observed magnetic behavior has been explained by Clar's rule of π electron.^48^ It is also known that substituting carbon atoms by nitrogen atoms in order to inject extra lone pairs to the system^49^ or the selective introduction of particular atomic-scale defects such as vacancies^50,51^ can be utilized to modify the electronic and transport properties of graphene.^52,53^ For instance, vacancies are associated with carbon magnetism.^54,55^ Among various introduced vacancy defects in graphene, nitrogen-doped vacancies, where carbon atoms are replaced by nitrogen atoms around the vacancy defect, are the most favored ones.^56–61^

Here, we propose a structurally simple, yet functionally efficient, spin filter composed of a ZGNR with embedded nitrogenated divacancy as a new promising building block to design and construct spintronic devices conceivable to be fabricated by simple synthetic methods.^59^ We show that while the proposed system with a robust FM configuration exhibits perfect half metallic behavior in the absence of mostly employed TM atoms, half metallicity in the electronic structure of the suggested system can also be obtained by the incorporation of a wide range of adatoms including semimetals, basic metals, and TMs. Our calculations indicate that FM ordering is the robust magnetic configuration of the suggested system. We also show that a two probe system, composed of the introduced half metallic structure, provides a perfect spin selective current with 100% spin filtering efficiency (SFE) within a practically feasible bias window.

## 2. Computational methods

Our first-principles calculations are performed based on density functional theory (DFT). The Vienna *ab initio* simulation package (VASP)^62^ is employed for geometry relaxations and investigation of the electronic and magnetic structures of the systems. The exchange–correlation effects are treated within the form of the generalized gradient approximation (GGA) of Perdew, Burke, and Ernzerhof (PBE).^63^ The electron–ion interactions are described by the plane-augmented wave (PAW) method and the Kohn and Sham orbitals are expanded in a plane wave basis set.^64^ A 400 Ry cutoff energy for the grid-mesh is employed in the calculations. A *k*-point mesh of 1 × 1 × 64 was employed along the *x*, *y*, and *z* directions. All the structures are fully relaxed until energy and forces are converged to 10^−5^ eV and 0.01 eV Å^−1^, respectively. To investigate the transport characteristics of the systems, DFT combined with nonequilibrium Green's function (NEGF)^65–68^ is employed. The spin-dependent transmission is given by

1 *T*_σ_(*E*, *V*_b_) = Tr[*Γ*_L_*GΓ*_R_*G*^†^]

where Tr denotes the trace, *Γ*_L/R_ = *i*[*Σ*_L/R_ − *Σ*^†^_L/R_] with *Σ*_L/R_ is the self-energy of the left/right electrode, and *G* = [*E* − *H* − *Σ*_L_ − *Σ*_R_]^−1^ represents Green's function with the scattering region Hamiltonian *H*. Within Tr[], all quantities implicitly depend on the energy (*E*), the bias voltage (*V*_b_) and the spin σ. The current is calculated using the Landauer–Büttiker formalism:2
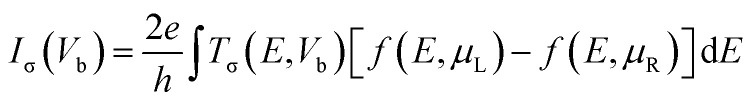
where *f*(*E*, *μ*_L/R_) is the Fermi–Dirac function with the associated chemical potential *μ*_L/R_ = *E*_F_ ± *V*_b_/2 that is a shifted value relative to the Fermi level of neutral system *E*_F_.

## 3. Results and discussion

**Fig. 1:**
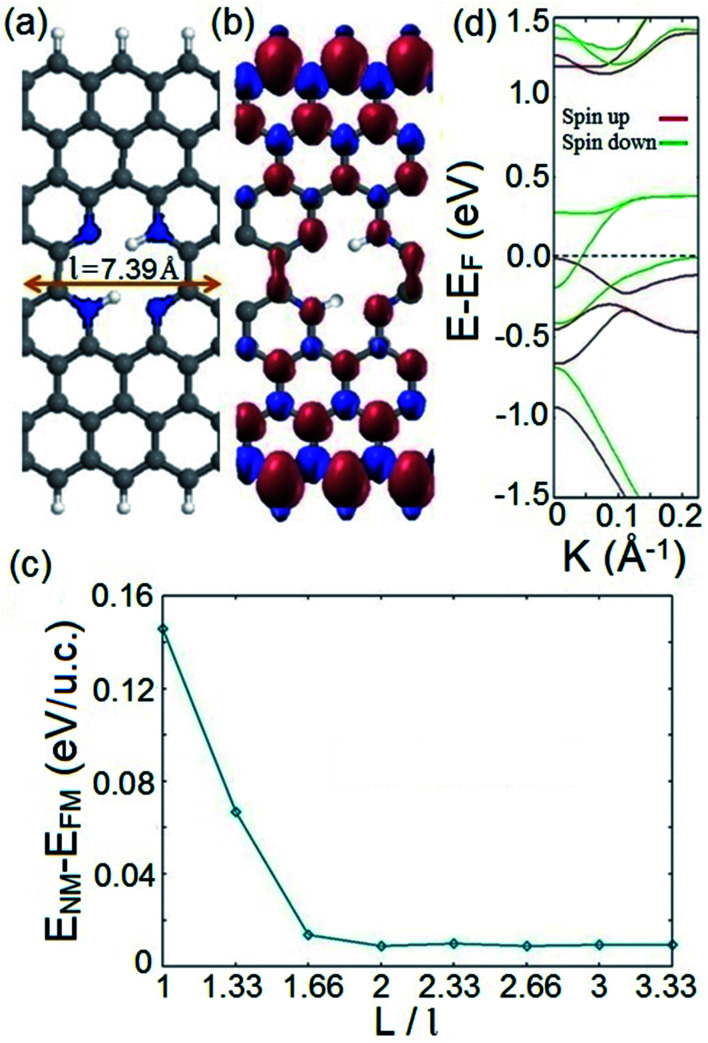
(a) Schematic illustration of the proposed spin filter with the smallest periodicity distance of *l* = 7.39 Å. C; gray, N; blue, and H; white. (b) Spin density distribution map of the depicted system. Spin up; purple, spin down: maroon. (c) Energy difference between the nonmagnetic (*E*_NM_) and ferromagnetic (*E*_FM_) configurations of the system as a function of the periodicity distance (*L*/*l*). (d) Spin resolved band structure of the depicted system. The Fermi level is shifted to zero and indicated by the black dashed line.

Fig. 1a presents an exemplary structure of the proposed spin filter system which is an *n* = 8 ZGNR (*n* represents the number of dimer lines along the width of the ZGNR) with an embedded nitrogenated divacancy. The depicted structure possesses the smallest possible periodicity length of *l* = 7.39 Å as indicated in Fig. 1a. It should be noted that the illustrated structure in Fig. 1a is energetically the most favored configuration among four possible structures of the proposed system in terms of the hydrogenation of divacancy (see Fig. S1 along with Table S1 in the ESI†). Since ZGNR host spin-polarized electronic edge states and vacancies can induce magnetism under appropriate conditions,^69–71^ it is worthy to investigate the magnetic configuration of the proposed spin filter system. To this end, we calculate and plot spin densities throughout the suggested structure in its final magnetic ordering, as depicted in Fig. 1b. Our calculations show that regardless of different initial spin orientations such as FM or antiferromagnetic (AFM) ordering between opposite edges, the converged magnetic ordering after self-consistent iterations is always FM. This is a remarkable finding because although the AFM ordering in an atomically straight ZGNR without doping is known to be the ground state, it is not robust against variation of different parameters such as thermal energy, carrier doping, or configurational variations. Therefore, it would be advantageous from the practical spintronics viewpoint to design and provide a ZGNR based spintronic system with a solid magnetic configuration. Our calculations indicate that the favored FM configuration of the system stays preserved for various widths of the employed ZGNR.

It would be useful from the application view to ensure that the FM ordering stays the favored magnetic configuration of the ground state of the system while the distance between adjacent divacancies changes. This can simulate a practical situation in which the distribution of divacancies along the ZGNR might be non-uniform. To this end, we perform spin-polarized total energy calculations for both the FM and nonmagnetic (NM) phases of systems with different periodicity (vacancy–vacancy) distances; *L*. Fig. 1c illustrates the energy difference between the NM and FM phases of the system as a function of a defined periodicity length (*L*/*l*). It is deduced from Fig. 1c that regardless of various distributions of divacancies along the ZGNR, FM ordering stays the favored lowest energy magnetic configuration of the suggested system. We also investigate the effect of the position of the divacancy with respect to the edges of the ZGNR on the magnetic ordering of the proposed system. The calculated spin density distributions for structures with divacancies at different positions are represented in Fig. S2.† It shows that the FM ordering of the proposed spin filter in its lowest magnetic configuration is preserved for various positions of the embedded vacancy.

We now investigate the electronic structure of the proposed system. The corresponding band structure of the depicted system in Fig. 1a is represented in Fig. 1d. It explicitly shows that a dispersive band line of down spin component crosses the Fermi level while there is an indirect band gap of 1.2 eV for spin up energy states near the Fermi level. This indicates that electrons with down spin exhibit metallic behavior, while spin up states possess a semiconducting feature. The obtained result is noteworthy because unlike previously studied systems, where half metallic behavior in introduced structures originated from the d electrons of the incorporated TMs,^11,72–74^ here, the suggested system exhibits half metallic behavior in the absence of d electron atoms. Hence the associated disadvantages of utilizing d electron elements are avoided in our proposed half metal system. This is a promising feature since it makes the introduced system a qualified candidate for fabricating a structurally simple and practically feasible spin filter device with a robust FM ordering.

**Fig. 2:**
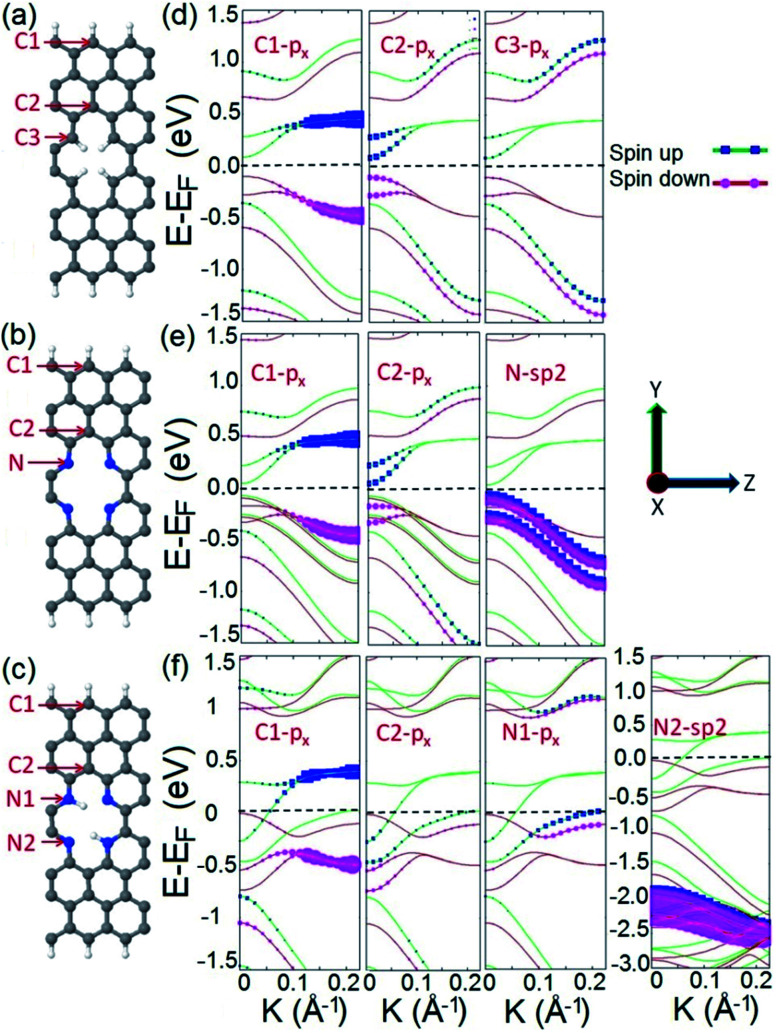
Schematic illustration of (a) C4H4, (b) N4, and (c) N4H2 systems. C; gray, N; blue, and H; white. (d–f) Orbital resolved band structures of C4H4, N4, and N4H2 structures respectively. The Fermi level is shifted to zero energy and indicated by the black dashed line.

To elucidate the origin of the observed half metallic characteristic of the proposed spin filter, we calculate and compare the orbital resolved electronic structures of three systems: C4H4, N4, and N4H2 as illustrated in Fig. 2a–c. As presented in Fig. 2d, the C4H4 system possesses a spin polarized band diagram mainly formed by the p_*x*_ orbitals of carbon atoms of the edges of the ribbon and divacancy (atoms C1, C2, and C3) in the vicinity of the Fermi level. This is in agreement with previous studies where it has been shown that divacancy defects presented in ZGNRs give rise to localized π states with energy close to the Dirac point^75^ which may interact with those originating from the zigzag edges of ZGNRs and lead to spin effects and ribbon magnetization.^76^ The N4 system possesses a similar band structure to C4H4 with four additional hybrid sp^2^ occupied energy bands appearing right below the Fermi level which originated from the additional electrons of the lone pairs of N atoms (Fig. 2e). This means that divacancy provides two independent contributions in the electronic structure of the N4 system; one from the occupied localized π states similar to that of C4H4 and the other from unsaturated σ bonds.

The electronic structure of the N4 system is modified when two diagonally facing nitrogen atoms in divacancy are hydrogenated to form the N4H2 frame. As Fig. 2f illustrates, the band structure of the N4H2 system is different from those of C4H4 and N4 in which it exhibits perfect half metallicity. The advent of the half metallic characteristic in the electronic structure of the N4H2 system can be explained as follows: in the N4 system, there are localized lone pair electrons of nitrogen atoms which can form σ bonding by the hydrogenation of N atoms (or incorporation of a dopant into divacancy as we will show later in this work). This means that in the N4H2 system, the hydrogenation of two nitrogen atoms not only forms σ type N–H bonding, but also injects two additional electrons into the electronic structure of the system. These additional electrons are supposed to reside on the π* antibonding orbitals.^77^ This enables the electrons to be transferred to the bulk carbon atoms, occupy the energy states near the Fermi level, and hence cause half metallicity. Our result is consistent with the most stable FM state of pristine ZGNRs for a wide range of carrier doping,^78^ where the doping is achieved by hydrogenation or adatoms in our system.

**Fig. 3:**
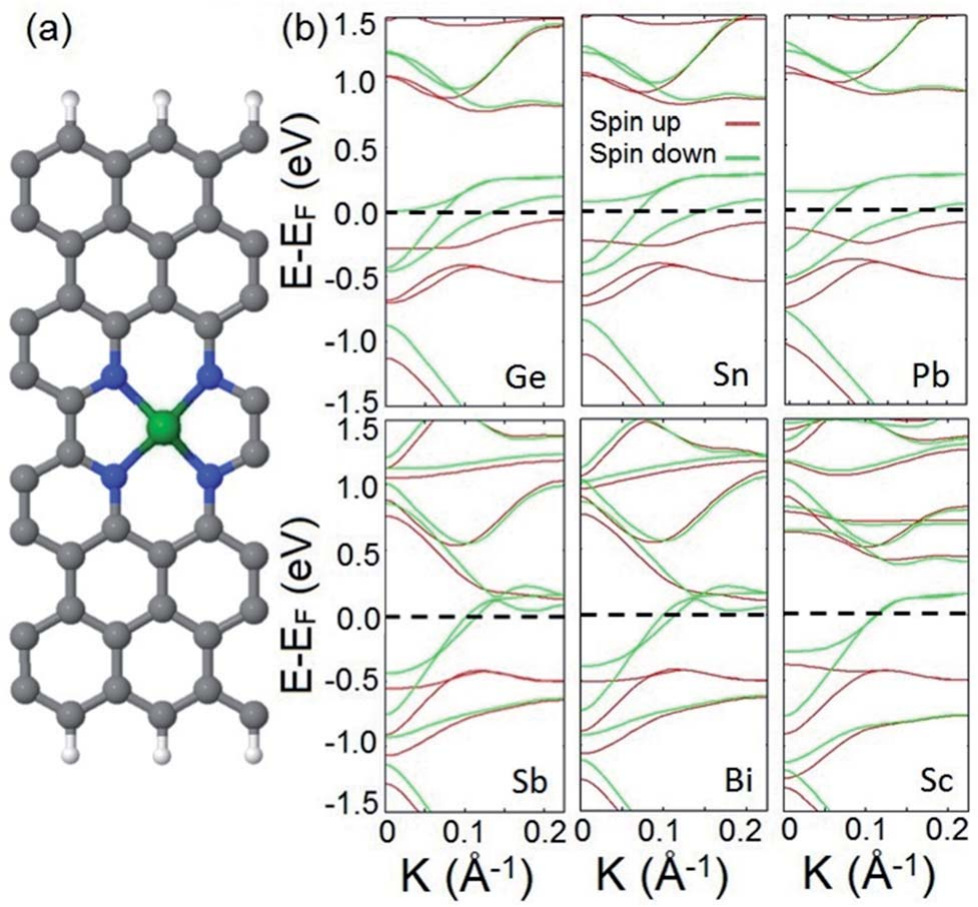
(a) A typical structure of the doped system. C; gray, N; blue, H; white, and doped metal; green. (b) Spin resolved band structures of the doped system with Ge, Sn, Pb, Sb, Bi, and Sc adatoms. The Fermi level is shifted to zero energy and indicated by the black dashed line.

We also study the incorporation of adatoms within divacancy and investigate the appearance of half metallicity in the electronic structure of the provided system. To this end, we choose a set of adatoms including Sn, Pb, and Bi from basic metals, Si, Ge, and Sb from metalloids, and Sc as a TM atom. A typical doped structure is illustrated in Fig. 3a. In the first step, we calculate the adsorption energy (*E*_ads_) of each adatom to the frame to verify the energetic stability of each adatom–frame system. The following formula is used to calculate the adsorption energies:

3 *E*_ads_ = *E*_atm+N4_ − *E*_N4_ − *E*_atm_

where *E*_atm+N4_ is the total energy of the optimized doped frame, *E*_N4_ is the optimized equilibrium energy of the N4 system, and *E*_atm_ is the energy of the isolated atom. The obtained *E*_ads_ values are summarized in Table 1. It is clear from the magnitude of the calculated *E*_ads_ that there is a covalent bonding character between adsorbates and divacancy which facilitates their interaction. It should be noted that although varied graphene nanoribbons impact the binding energy of dopants to some extent, the formation of the provided doped system is stable enough to be utilized in practice such as in spintronic applications as well as in catalytic reactions.^79^ Our calculations also show that similar to the case of the introduced structure in Fig. 1a, FM ordering is the robust favored magnetic configuration of the studied doped structures.

**Table 1:** Adsorption energy (*E*_ads_) of different adatoms to divacancy

Adatom	*E*_ads_ (eV)
Ge	−6.50
Sn	−4.51
Pb	−3.83
Sb	−4.25
Bi	−3.26
Sc	−7.39

Next, we calculate and analyze the electronic structures of the introduced doped systems. The calculated band structures are illustrated in Fig. 3b. It should be noted that to calculate the band structure of the Sc doped structure, the effect of strongly correlated electrons is included (see the ESI† for computational details). The plotted band diagrams explicitly show that all suggested doped systems expose perfect half metallic behavior. This indicates that non-TM atoms can participate in half metallicity as well as TMs.

To confirm that the above mentioned explanation for the origin of half metallicity in the proposed spin filter applies for the doped system as well, we calculate and plot the orbital resolved band structures of the Ge doped system as an example of a doped system and compare it with the one plotted for the hydrogenated divacancy. The calculated orbital resolved band diagrams of the Ge doped structure are shown in Fig. S3.† Comparing the fat-band diagrams of the two systems clearly shows that the incorporation of adatoms into the divacancy also provides additional electrons which occupy the π* antibonding orbitals of the system, and interact with electrons of edge carbon atoms and consequently leads to manifestation of half metallicity. The remarkable finding of the obtained results is that in our introduced system, besides the d orbital electrons of TMs, the p orbital electrons of the incorporated non-TM atoms can also give rise to robust magnetism and lead to half metallicity (the calculated magnetic moments of p orbital electrons are presented in Table S2 in the ESI†).

Although the introduced system having divacant sites exhibits half metallic behavior in the absence or presence of embedded metals, we show below that introducing certain adatoms into the system results in the enhancement of the spin polarized current of the designed spin filter device.

**Fig. 4:**
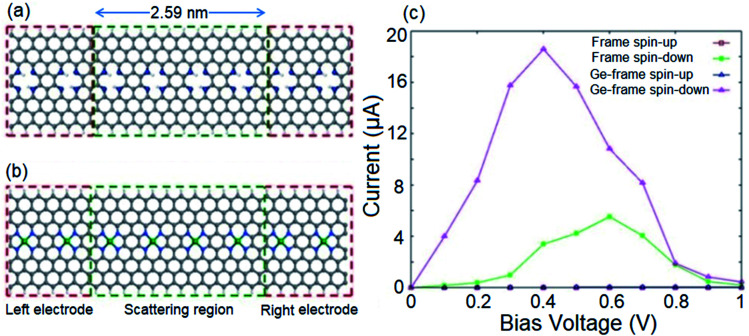
Schematic illustrations of the proposed two-probe systems for (a) hydrogenated and (b) Ge-doped systems. *n* = 8 ZGNR is used for both scattering and semi-infinite electrode regions. (c) Spin resolved current–voltage profiles of hydrogenated and Ge-doped transport systems.

Given the intrinsic half-metallicity of hydrogenated and metal doped configurations, we investigate the spin transport features under a finite bias voltage towards feasible spin filter devices. The schematically depicted structures in Fig. 4a and b, constructed using an *n* = 8 ZGNR, represent the utilized two-probe systems to study the spin resolved transport properties of the hydrogenated and Ge doped systems. The obtained current–voltage (*I*–*V*) profiles of both transport systems are illustrated in Fig. 4c. It should be noted that the bias voltage (*V*_b_) can be chosen with respect to the band gap discrepancies of spin components to obtain the desired proportion of spin filtering. Here, the *V*_b_ range of 0 to 1 V would be an appropriate bias window to attain the desired spin filtering effect. The selected bias window is also an appropriate choice from the view point of application, since it is not that high to modify the geometrical structure of the device.^80^ It follows from Fig. 4c that in the range of applied *V*_b_, the spin down component in the *I*–*V* profiles of both systems show a nonzero current, while the transport channel for carriers with up spin is blocked. Although both systems provide similar spin polarized *I*–*V* profiles, however it should be noted that the magnitudes of the provided currents by two systems are different. It is shown in Fig. 4c that the Ge incorporated transport system provides a larger spin polarized current compared to the hydrogenated system. The reason for the enhancement in current for the metal doped system can be explained by comparing the band structures of the two systems represented in Fig. 1d and 3b. One can see that the metal doped system provides more transport channels in the vicinity of the Fermi level compared to the hydrogenated structure.

Fig. 4c also shows that for both systems, while the spin up current stays negligible by increasing *V*_b_, the spin down current increases and reaches its maximum value of 5.5 μA at *V*_b_ = 0.6 V for the hydrogenated structure and 18.6 μA at *V*_b_ = 0.4 V for the Ge-doped transport system. However, for further increase of *V*_b_, the spin down current becomes suppressed due to the presence of an energy gap near 0.5 eV. In the case of *V*_b_ values greater than the energy gap for the spin up component, the spin up current becomes non-zero. This indicates that in addition to spin filtering characteristic, the proposed spin filter device also exhibits negative differential resistance (NDR).^81^

**Fig. 5:**
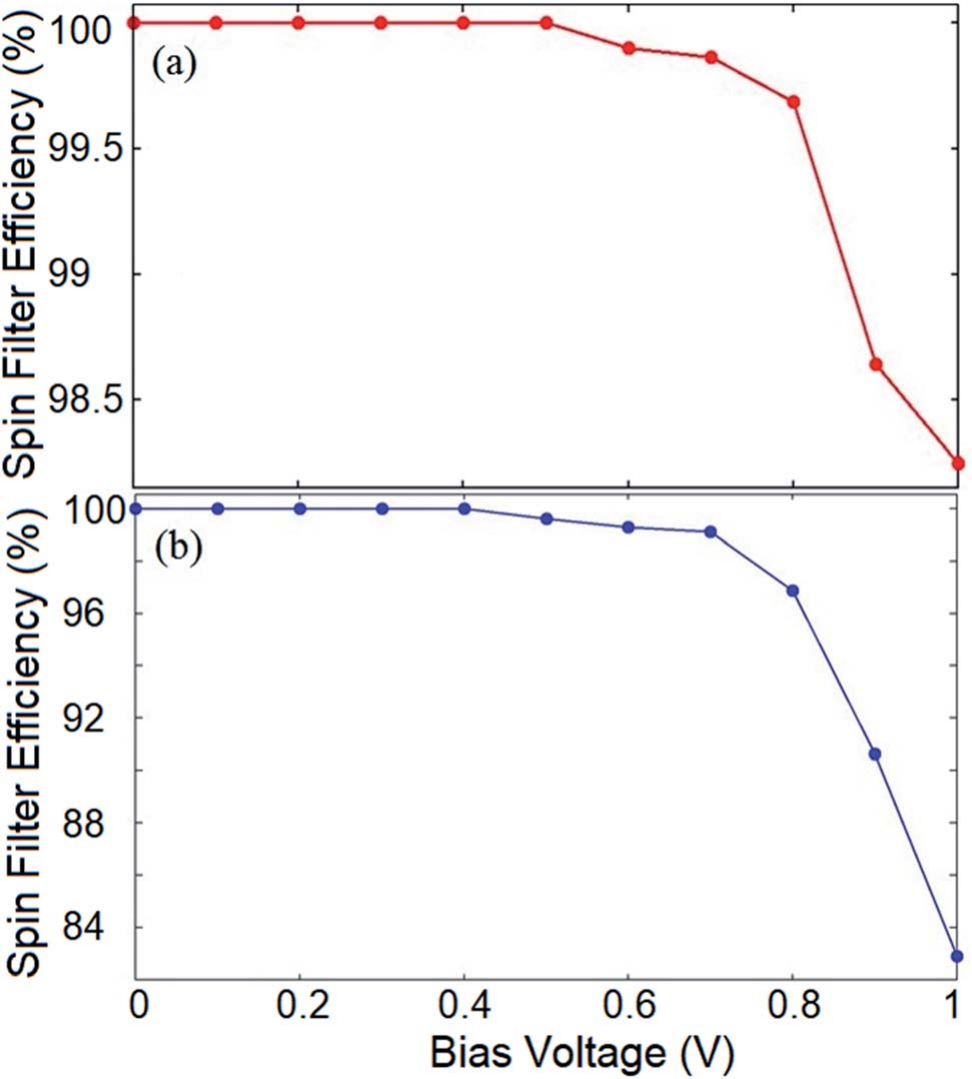
Spin filter efficiency of the (a) hydrogenated, and (b) Ge doped frame.

To evaluate the spin filtering capability of the proposed device, we calculate the SFE of the system for both the introduced configurations. The SFE at a bias voltage is defined as:

4 SFE = |(*I*_↑_ − *I*_↓_)/(*I*_↑_ + *I*_↓_)| × 100

where *I*_↑_ and *I*_↓_ refer to the current in the up and down spin channels, respectively. At zero bias, the SFE can be calculated by replacing the current values with the corresponding transmission coefficients. The larger SFE values indicate a better spin filtering effect. If the SFE value of the device approaches 100%, the device can serve as a perfect spin filter. Fig. 5a illustrates the variation of the calculated SFE for the hydrogenated spin filter device depicted in Fig. 4a. It shows that the device exhibits a fully spin filtered *I*–*V* profile for *V*_b_ values up to 0.5 V. For higher *V*_b_, the SFE decreases and drops to ∼98% at *V*_b_ = 1 V which is still a significant efficiency. The SFE of the Ge doped spin transport system is represented in Fig. 5b. One can see that the Ge doped spin transport system also provides 100% spin filtered current for *V*_b_ values up to 0.4 V while it decreases to 82% at *V*_b_ = 1 V.

It should be noted that for both the hydrogenated and doped structures, the outcome would be different at higher *V*_b_ values where the spin up and down states may possess band gaps which are smaller than the bias window, and therefore, the current would be non-zero for both spin components. This provides a remarkable opportunity to employ the proposed system as a nano-size on/off spintronic switch where the spin filtering characteristic of the device can be tuned by the applied bias voltage.

Although the spin anisotropy plays a crucial role for the long range spin ordering in nanostructures, it is worth noting that according to the Ising model of one-dimensional spin chains, such as our proposed system, the spin ordering is allowed only for a zero temperature limit and the spin susceptibility diverges around *T* ∼ *J*/*k*_B_ where *J* is the spin exchange constant and *k*_B_ is Boltzmann's constant.^82^ Nevertheless, the spin–spin correlation length, *ξ* ∼ −ln[tanh(*J*/*k*_B_*T*)] with the exchange constant of *J* ∼ 0.1 eV per u.c. from our calculations, becomes longer than 100 units at room temperature, which indicates a probable ordering in a finite length ribbon shorter than 100 nm. For more discussion on the temperature dependence, further separate studies are needed.

## 4. Conclusion

In summary we have introduced a high performance and structurally simple graphene based spintronic device with technically feasible characteristics. Utilizing first principles calculations, we have shown that a ZGNR with a hydrogenated CN4 divacancy exhibits perfect half metallic behavior. Our calculations indicate that the proposed system possesses a robust FM ordering, which is steady against configurational variations of the system including the change in the width of ZGNR, and the distribution of divacancy defects over the system. The advent of magnetism in the proposed structure indicates that unlike previously studied graphene based spin filters, the incorporation of TM adatoms into the suggested system is not essential to attain half metallicity. Nevertheless, we have studied the embedding of different dopants including semimetals, metalloids, and TMs into the divacancy and its effect on the spin filtering effect of the proposed system. We have shown that the provided metal doped systems also exhibit steady FM ordering with perfect half metallicity. This means that in the proposed system, magnetism originated from p orbital metals also, as well as commonly employed d orbital TMs. We have also investigated the transport properties of spin filter devices constructed using introduced hydrogenated or metal doped systems. We have demonstrated that both the designed spin filters can generate a fully spin polarized current, where the metal doped transport system provides a larger current magnitude compared to the one obtained from the device constructed utilizing a hydrogenated frame. We have shown that both electron transport structures possess a SFE of 100% for practically feasible bias voltages. It has also been shown that aside from the spin filtering effect, the proposed structures exhibit NDR as well. Possessing all these functional and robust characteristics, our proposed spin filter system would be a promising candidate to achieve a practically feasible atomically thin spintronic device.

## Conflicts of interest

There are no conflicts of interest to declare.

## Supplementary Material

NA-002-D0NA00652A-s001
