# Supplementary material for: A high performance N-doped graphene nanoribbon based spintronic device applicable with a wide range of adatoms
Source: Nanoscale Adv. 2020 Nov 9;2(12):5905–11. doi: 10.1039/d0na00652a (PMC9419213; doi:10.1039/d0na00652a)
Supplement: NA-002-D0NA00652A-s001 [file NA-002-D0NA00652A-s001.pdf]

Electronic Supplementary Material (ESI) for Nanoscale Advances.
This journal is © The Royal Society of Chemistry 2020

Electronic Supplementary Information for

"High performance N-doped graphene nanoribbon based spintronic device applicable with a wide range of adatoms"

M. Reza Rezapour[a], Geunsik Lee[a,b], and Kwang S. Kim[a*]*

[a]*Center for Superfunctional Materials, Department of Chemistry, Ulsan National Institute of Science and Technology (UNIST), Ulsan 44919, Korea.*

[b]*Department of Chemistry, Ulsan National Institute of Science and Technology (UNIST), Ulsan 44919, Korea.*

*kimks@unist.ac.kr *gslee@unist.ac.kr

Energetic stability of the system

Here we aim to find the most stable structure of the proposed spin filter device in terms of the H-passivation of the embedded divacancy. To do this, we employ four different frames in which each has a different H-passivation of divacancy (Fig. S1a-e). All of the structures are fully relaxed under the conditions mentioned in the main text. We then calculate the total energy (E_{tot}) and the formation energy (E_{form}) of each relaxed structure. E_{form} is calculated using the following equation:

$$E_{form} = E_{tot} - E_{ZGNR} + N_C\mu_C - N_N\mu_N - N_H\mu_H$$

Where E_{ZGNR} is the total energy of pristine ZGNR. N_C is the number of removed carbon atoms from vacancy while N_N and N_H represent the total number of nitrogen and hydrogen atoms in the proposed system respectively. μ_C, μ_N, and μ_H denote chemical potentials of carbon, nitrogen, and hydrogen atoms, respectively. The chemical potential of carbon (μ_C) is defined as the total energy of graphene per carbon atom, and the chemical potentials of nitrogen and hydrogen atoms are taken as one-half of the total energy of the N_2 and H_2 molecule in the gas phase respectively.

The obtained E_{tot} and E_{form} values for each structure are represented in Table S1. Comparing the E_{form} values indicates that the frame with divacancy in which two diagonally positioned N atoms are H-passivated (the structure c) has the highest energetic stability among the four possible configurations of the proposed system.

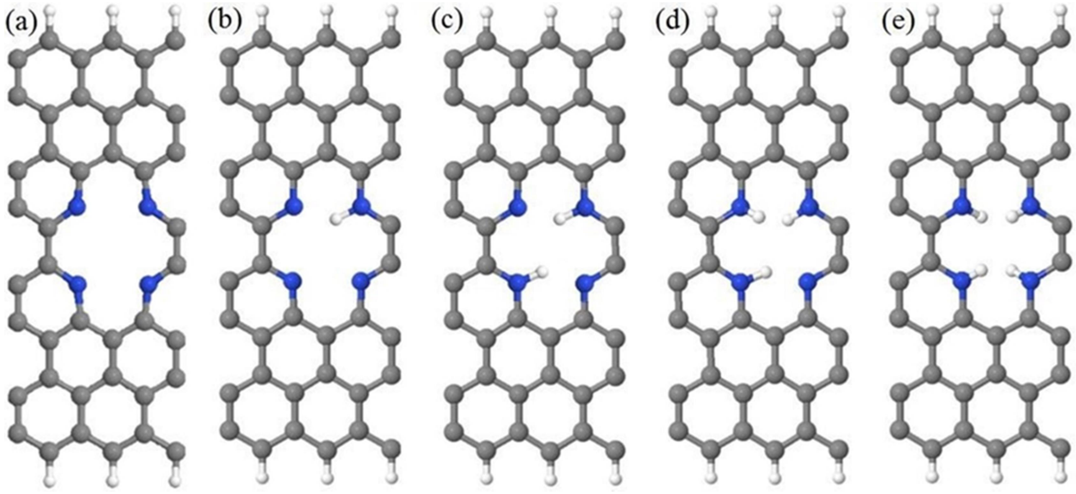

Fig. S1: (a) N4, (b) N4H1, (c) N4H2, (d) N4H3, and (e) N4H4 systems. All the structures are fully relaxed. C; gray, N; blue, and H; white.

Table S1: Total energy (E_{tot}) and formation energy (E_{form}) of each possible configuration of the frame.

Structure	E_{tot} (eV)	E_{form} (eV)
N4	-419.715	4.02
N4-H1	-424.85	2.25
N4-H2	-429.75	0.74
N4-H3	-432.61	1.25
N4-H4	-435.33	1.91

The effect of the width of ZGNR and the position of divacancy on the magnetic configuration of the system

To investigate the effect of the width of ZGNR on the magnetic ordering of the system, we calculate the total energy of the system for its initially ferromagnetic (E_{FM}) or antiferromagnetic (E_{AFM}) oriented configurations. We perform the calculation for four different widths, n = 4, n = 5, and n = 6, and n = 8 where n stands for the number of C-C dimers along the width of ZGNR. Our obtained results confirm that the system in its final magnetic configuration possesses the same total energy regardless of its initial magnetic configuration. We also discuss the effect of the position of divacancy on the magnetic configuration of the frame. Fig. S2 illustrates the final magnetic orderings of frame for its three different configurations in terms of the positions of divacancy. While the center of divacancy is located at the same distance from both edges of frame in Fig. S2-a, the symmetry is broken for the structures represented in Fig. S2-b and c, where the center of divacancy is closer to one edge than the other. However, the calculated spin density distributions over the demonstrated three structures reveal that regardless of the position of divacancy, the magnetic configuration of the frame is always FM as shown in Fig. S2.

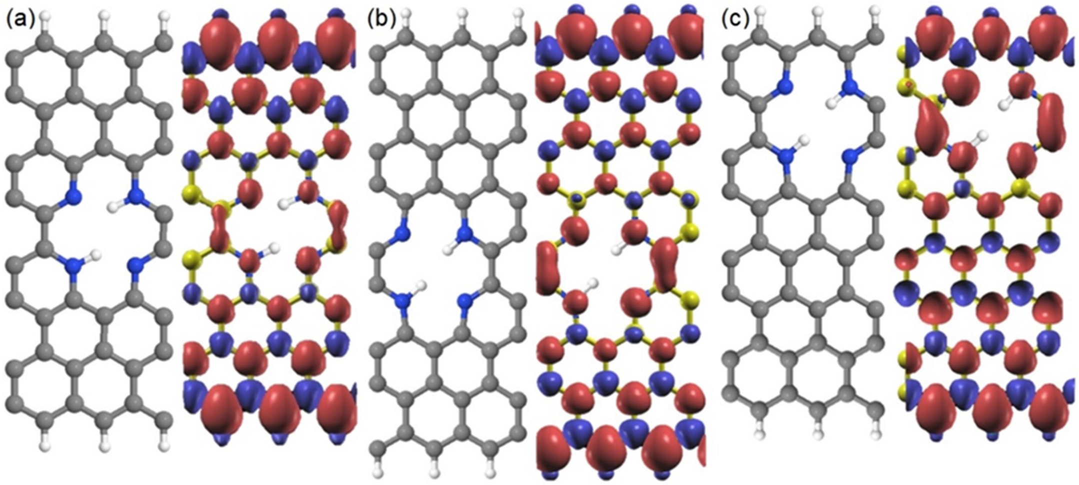

Fig. S2: (a)-(c) Three different structures of the proposed system in terms of the position of divacancy and their corresponding spin density distribution maps. C; gray and yellow, N; blue, and H; white. Spin up; purple, spin down; maroon.

Applying the effect of strongly correlated electrons to the band structure calculation of Sc doped system

It would be informative to explore the half metallic behavior of the proposed structure with TM dopants. In order to apply the effect of strongly correlated electrons, the onsite Coulomb (U) and exchange interaction (J) parameters, are included in the calculation. In a simplified rotationally invariant formulations,[1,2] U and J are combined to form an effective parameter $U_{eff} = U - J$. We here take a value of $U_{eff} = 3.0$ eV ($J = 1.0$ eV) for TM d orbitals.[3,4] The obtained band structure, represented in Fig. 3b, shows that the incorporation of Sc in the suggested frame also leads to half metallic behavior of the system.

Orbital resolved band structure of the Ge doped system

We calculate and plot the orbital resolved band structure of the Ge doped frame (Fig. S3) to investigate the half metallicity in the doped frame. Only fat band diagrams of carbon atoms with main contribution in forming band lines within the chosen energy window are represented. It is deduced from the depicted orbital resolved diagrams that the p orbital electrons of Ge adatom contribute to the observed magnetism in the electronic structure of the doped frame and lead to

half metallicity. The calculated magnetic moments of p orbital electrons in the doped systems by adatoms indicated in Table 1 are presented in Table S2.

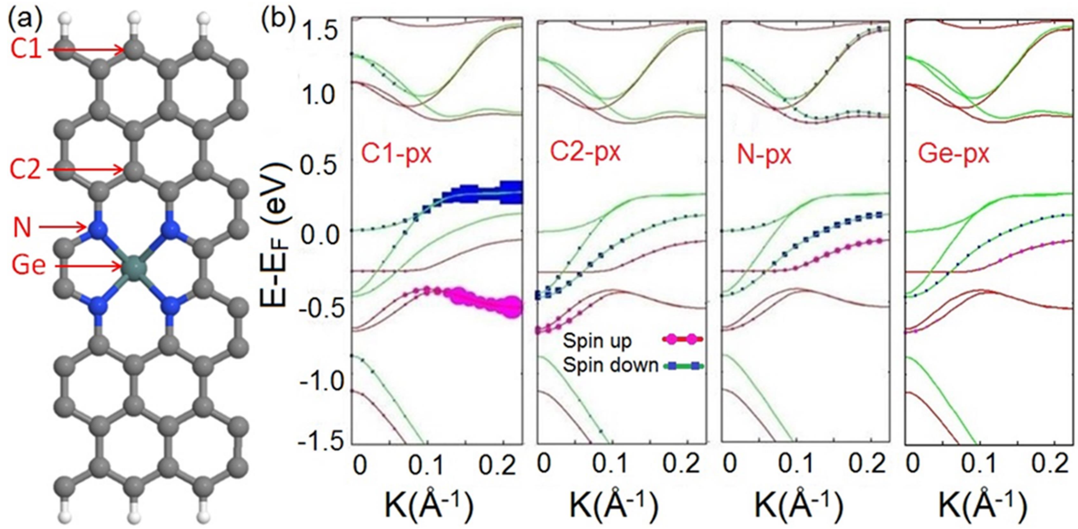

Fig. S3: (a) The structure of Ge doped system. C; gray, N; blue, H; white, and Ge; green. (b) Contributions of p orbitals of labeled carbon atoms, nitrogen atom, and Ge atom in Fig. S3a to the electronic structure of the Ge doped system.

Table S2: Magnetic moments of p orbital electrons in the doped systems

Structure	Magnetic moment (bohr)
Ge-doped	1.97
Sn-doped	1.991
Pb-doped	0.90
Sb-doped	1.00
Bi-doped	1.00
Sc-doped	0.98

References

1 S. S. Li, Y. P. Wang, S. J. Hu, D. Chen, C. W. Zhang and S. S. Yan, *Nanoscale*, 2018, **10**, 15545-15552.

2 M. Li, D. Zhang, Y. Gao, C. Cao and M. Long, *Org. Electron*., 2017, **44**, 168-175.

3 V. I. Anisimov, J. Zaanen and O. K. Andersen, *Phys. Rev. B*, 1991, **44**, 943-954.

4 V. I. Anisimov, F. Aryasetiawan and A. I. Lichtenstein, *J. Phys.: Condens. Matter*., 1997, **9**, 767-808.
